# “We Need Other Human Beings in Order to be Human”: Examining the Indigenous Philosophy of Umunthu and Strengthening Mental Health Interventions

**DOI:** 10.1007/s11013-020-09692-4

**Published:** 2020-11-19

**Authors:** Jerome Wright, Janaka Jayawickrama

**Affiliations:** 1 Department of Health Sciences, University of York, Seebohm Rowntree Building, Heslington, York YO10 5DD UK; 2 Centre for Community Wellbeing, Department of History, College of Liberal Arts, Shanghai University, Shanghai, China

**Keywords:** *Umunthu*, Malawi, Mental distress, Collaboration, Health Surveillance Assistants

## Abstract

This paper examines how cultural, historical and contemporary perspectives of mental health continue to inform ways of understanding and responding to mental distress even under the biomedical gaze of the Movement for Global Mental Health (MGMH). Based on experiences in Malawi, the authors explore three prominent interventions (practical support, counselling and support groups) employed by village health workers within a mental health task-shifting initiative and reveal how the ancient philosophy of *Umunthu* with its values of interconnectedness, inclusion and inter-relationships informs and shapes the direction of these interventions. Practical support is marshalled through traditional village structures, counselling provides advice and an encouragement to hope, and support groups provide a place for emotional exchange and a forum for the enactment of values, reflection and reinforcement of *Umunthu*. What are pronounced as biomedical psychosocial interventions are in fact the delivery of culturally embedded therapeutic approaches. Historical and socio-political evidence is offered to explain the dominance of biomedical perspectives and the HSAs’ responses and a call is made for a transformation of MGMH to embrace rich philosophies such as *Umunthu* and enact respectful, inclusive and democratic values to enlist collaborations between equals to develop relevant and effective knowledge and local responses to mental distress.

## Introduction

The aim of this article is to show how cultural, historical and contemporary perspectives of mental health beyond the Movement for Global Mental Health (MGMH) inform ways of understanding and responding to mental distress. Through the case of a mental health task-shifting initiative in Malawi as the central theme, this article examines underlying indigenous understandings that underpin positive ways of responding to peoples’ experience of distress and the different lenses through which these interventions can be understood. In this article, the use of the word indigenous means the worldviews developed at a specific society, by people through their generational, geographical and traditional experiences. The word ‘distress’ has been adopted to describe a cumulative experience that biomedicine would term a mental health problem or mental disorder. Although it is accepted that the term is unspecific and can denote a range and severity of experience, it is also used here to acknowledge social conceptions of a phenomenon where local classifications do not differentiate biomedical categories (for instance, between disability and mental health problems). Equally, since all cultures define nosologies of cognition, reasoning, behaviour and social interaction, the terms mental illness and disorder are also used within the paper to denote such phenomena in its widest sense, despite its conspicuous link to biomedicine.

Specifically, the paper explains how the African philosophy of *Umunthu* continues to resonate as a contemporary, sophisticated yet pragmatic approach, despite—and in fact under the gaze of—western biomedical approaches in Southern Malawi. Suggestions will be advanced as to the implications of this for MGMH approaches and the importance of developing an inclusive, respectful and democratic approach to understanding and responding to distress.

## Background

Life is uncertain and impermanent (Kleinman 2019; Lindekens and Jayawickrama 2018). In response, all societies have developed their own epistemologies and approaches to dealing with these realities which are specific to the social, political, cultural, economic and environmental contexts of each society (Jayawickrama 2018; Scott 1979). And ‘mental health’ itself is a concept with its own socially produced history (Bertolote 2008).

Since their first global summit in Athens in 2009, the Movement for Global Mental Health (MGMH) aims to improve the availability, accessibility and quality of services for persons with mental disorders by scaling up services through the fundamental principles of scientific evidence and human rights (MGMH 2020). Yet this declaration presents a huge incongruity by on the one hand drawing much needed attention to the distress and suffering of a large proportion of vulnerable people, but at the same time presenting one medicalised worldview through which to frame these experiences (Clark 2014).

Aside from difficulties with the scientific validity of this approach, where particular ‘life problems’ are defined as mental health problems (Summerfield 2008) and are seen as most effectively responded to by ‘health services’ rather than wider social, welfare and educational sectors (WHO 2014), to assume that everyone experiences suffering in the same way has been deemed as morally wrong (Kleinman 2006). Specifically, the reluctance of biomedicine to acknowledge and explore ‘healing’ from other perspectives, because entertaining such notions “strips away the illusion that biomedical research, is the only scientific approach to healthcare problems” (Kleinman 1980:312).

For from the MGMH position (MGMH 2020), it is evident that there is at best a lack of understanding, or, at its worst, no acknowledgement that people in many countries outside Europe and North America have their own existing, sophisticated yet pragmatic approaches to dealing with life and suffering. ‘Civilizations’ as a series of global, historic and current entities are replete with examples of systematic approaches to peoples’ suffering. From Ayurveda (3300 BCE to 1300 BCE), Chinese Medicine (14th–11th centuries BCE), Persian Medical Sciences (224–651 CE) as well as other approaches in Africa (Lindekens and Jayawickrama 2018), explanations and approaches to mental health problems and challenges to the overall wellbeing of people have been developed. While the history of mainstream modern medicine can be traced to Hippocrates (400 BCE), the rise of this practice in Europe and North America only arose in the 19th Century and the contemporary concept of mental health, originating from the mental hygiene movement, was only instigated in 1908 (Beers 1937). Indeed, if health systems such as the Ayurvedic or Chinese Medical explanations on health, including mental health, or all other approaches are disregarded or erased from certain ‘histories’, it would be reasonable to ask how humanity survived the past 300,000 years before the “invention” of mental health concepts and interventions that the MGMH now advocate. But given this current ‘global’ emphasis, more specifically it may now be asked, what might be the impact of these biomedical assumptions and emphases on peoples’ experiences of ‘distress’ and on those charged with their support? Or, to what extent is the articulation of distress and the way that support is provided mediated through the lens of biomedicine or other understandings?

## Case from Malawi

Recent approaches to mental health in Malawi have aligned with the MGMH and placed an emphasis on reducing the ‘treatment gap’ through employing a combination of pharmacological and particular psychological interventions (WHO 2016). As has been shown, the deployment of pharmacological treatments and their political, socio-economic and physical effects and side effects have received often highly charged critiques, yet increasing access to such treatments has been seen as a major imperative for Malawi (Udedi 2016). However, aside from arguments as to their efficacy (overwhelmingly undertaken in high resource countries), the underlying assumptions of the ‘psychological interventions’ have, it seems, received far less attention. One initiative to promote mental health integration within primary health care in rural Malawi provides an interesting example of the deployment of such ‘psychological’ interventions and how, through these encounters, the interaction between different assumptions and understandings of distress and effective responses can be clearly illuminated. The first author of this article (JW), a white British nurse and researcher, has worked for the last fifteen years with Non-Governmental Organisations (NGOs) and Government sectors in Malawi on a number of HIV/AIDS and mental health initiatives—including leading the primary care mental health project described here. These experiences have offered the opportunity to gain an understanding of both formal and informal help-seeking and provision within Malawian life. A Sri Lankan by birth and disposition, the second author (JJ) has been collaborating with disaster, conflict and uneven development affected communities in Asia, Africa and the Middle East over the last 26 years. He was initially trained in an individual model of psychological care but soon realised that many communities value the collective rather than the individual. He engaged with refugees in Malawi to assess the mental health and wellbeing interventions during May to November 2006. In this, he closely collaborated with traditional healers in Malawi, with whom the refugees heavily drew upon to deal with mental health challenges and improve their wellbeing. Whilst immersed in the field during each project assignment, both the authors are ‘outsiders’ to Malawi and the continent of Africa. Instead, the authors collaborated with Malawian project partners and research colleagues for direction and in-depth discussion and on our own curiosity and reflexivity based upon years researching mental health and wellbeing in different cultures across Africa, Asia and the Middle East. That said, within explorations of ‘everyday life’, there may be advantages that an ‘outsider’ may have in discerning phenomena that can in turn be subjected to interpretive and collaborative discourse with ‘insiders’ and literature. Such reflective processes may be indicated in the fact that, while engaged with Malawi in separate projects and at different times, the authors’ independent conclusions about the importance of *Umunthu* to mental health and wellbeing became the foundation for this collaborative article. This unreservedly retrospective analysis of data deriving from project participant observation notes, survey and interview data and contemporary research literature places the method as accidental ethnography (Leviton, Carr-Chellman and Carr-Chellman 2017). The explicit focus on past information and experiences and, through reflexive discussion, to develop theory and practitioner knowledge, serves an ethnographic purpose to influence future directions – in this case, the strengthening of mental health support.

A district-wide task-shifting project utilised village-based health workers—Health Surveillance Assistants (HSAs)—to undertake mental health assessments, interventions and health promotion in an area previously renowned for its limited resonance of psychiatry (Steinforth 2009). Within the community, the identification and treatment of persons with *misala* (madness) or other mental health problems were managed as they have done for centuries through both family and community resources. Formal structures of traditional authorities, from healing approaches derived from Traditional African Religions and from Christian Churches provide the recognised care. Within the initiative, the determination of ‘mental illnesses’ was based on HSAs’ assessment of level of ‘distress’ and ‘risk’ (to themselves and others), and there was no attempt to offer a psychiatric diagnosis (Wright and Chiwandira 2016). The training of HSAs did, however, incorporate education on common mental health problems, psychosis, epilepsy and intellectual impairment; approaches to assessment of peoples thoughts, feelings and behaviour, their context, vulnerability and strengths; and interventions (‘responses’) in the form of listening, identifying the problem areas, sign-posting to further help locally and through government health services and providing emotional support individually (counselling) and group support. The key interventions, offering individual psychological and emotional support and mobilising community psychosocial resources, were essentially community based and matched psychosocial packages of care commonly designed for low resource settings (Lund, Tomlinson and Patel 2015).

From the evidence of engagement and outcomes, the project showed that HSAs were able to offer a practical and acceptable health model for mental health promotion and care and was seen as contributing to the evidence-base for the benefits of a biomedical approach. Yet closer analysis through a further qualitative study (Wright and Maliwichi-Senganimalunje 2019) revealed a more complex and nuanced picture. The pluralistic nature of mental health attributions and help seeking were clearly operating, with both patients and HSAs retaining a veiled and apparently ambivalent relationship with traditional forms of healing. Patients consulted traditional healers but were not inclined to disclose this to biomedical health professionals, yet health professionals themselves both accessed traditional healers for their own ills and felt the need to ignore or denigrate any patients’ use of traditional healing, even where interventions had proved a success (Wright and Maliwichi-Senganimalunje 2019). So given this pluralistic context, where different positions and approaches to healing coexist, it seems appropriate to look again at the ‘mental health interventions’ that the HSAs had employed in their work with patients in distress. The data reveal that three interventions in particular were most prominent:

1. Practical assistance mobilised through community structures and governance—e.g. keeping people safe from harm, tracing guardians, accessing food, shelter through chief/elders.
2. ‘Counselling’—e.g. providing advice on lifestyle (reducing drinking alcohol or smoking *chamba* (marijuana) and advice to gain spiritual support and guidance),
3. Encouragement to join support groups, community gatherings and activities.
  (Wright and Chiwandira 2016:593).

It was clear that each of these interventions can be articulated from a biomedical mental health perspective (WHO 2016) and derive from the range of ‘psychosocial mental health interventions’ consistent with a MGMH approach. However, given the pluralistic context that HSAs were a part, there may be something more to the prominence and repeated use of these interventions which suggest that the HSAs hold particular attachment and significance not fully explained by an allegiance to the biomedical model. Indeed, defining the activity of HSAs in these interventions through the lens of a global mental health psychosocial intervention may be a less than convincing description. Clearly, ‘psychosocial interventions’ are not exclusive to one epistemology and will carry different meaning and emphasis. Kleinman (1980) points to the interrelated nature of health care activities where the healing process (in this case particular ‘psychosocial interventions’) does not exist outside of a health system which includes an understanding of the illness experience and patient-practitioner transaction (p. 24). For, if the HSAs (and patients) were drawing on existing indigenous knowledge—whether from traditional structures or spiritual understandings—where does this come from and what informs and sustains this approach? How do these approaches resemble or differ from a biomedical approach? And, might the articulation of interventions through MGMH terminology and classifications be obscuring indigenous philosophies, ways of understanding distress and helping approaches much closer to home and which carry their own assumptions and interventions? A response must surely begin with an exploration of the existing philosophy of *Ubuntu* (or in Malawi, *Umunthu*) ubiquitous throughout the country and the rest of sub-Saharan Africa.

## Defining *Umunthu*

“A person is a person through other persons. None of us comes into the world fully formed. We would not know how to think, or walk, or speak, or behave as human beings unless we learned it from other human beings. We need other human beings in order to be human.”

Tutu, 2004:25.

The word *Umunthu* is derived from an Nguni (isiZulu) aphorism: *Umuntu Ngumuntu Ngabantu*, which can be translated as “a person is a person because of or through others” (Moloketi 2009:243; Tutu 2004:25–26). *Ubuntu* can be described as the capacity in an African culture to express compassion, reciprocity, dignity, humanity and mutuality in the interests of building and maintaining communities with justice and mutual caring (Khoza 2006:6; Luhabe 2002:103; Mandela 2006:xxv; Tutu 1999:34–35).

Samkange and Samkange (1980) highlight the three sayings of *Umunthu*. The first saying asserts that to be human is to affirm one´s humanity by recognising the humanity of others and, on that basis, establish respectful human relations with them. And the second saying offers that if and when one is faced with a decisive choice between wealth and the preservation of the life of another human being, then one should opt for the preservation of life. The third saying is a principle deeply embedded in traditional African political philosophy that says that the ruler owes their status, including all the powers associated with it, to the will of the people under them.

*Umunthu* can be considered as both an expressive account of value systems that operate across much of Sub-Saharan Africa as well as a normative philosophy of how people should relate to one another. These perspectives contain three points that are relevant understanding the conceptualisation of mental health and wellbeing in Sub-Saharan Africa.

1. Interconnectedness: *Umunthu* as a philosophy can only be operationalised through relationships. The expression, *I am because we ar*e, is the best example of this aspect. This points towards an individual’s sense of being cannot be separated from the family, community and social context. It also highlights the importance of a subjective and emotional appreciation of human experiences including disasters, conflicts and uneven development.
2. Inclusion: As a collective philosophy and operational methodology, *Umunthu* promotes the oneness with everyone and everything around the individual. In this realisation, compassion, care, respect and dignity are shared values. *Umunthu* advocates the moral value of the importance of collaboration in the face of crisis. It is important to understand that in a region where populations have experienced colonial looting, social, political, economic, cultural and environmental disorder, the collective action and mutual assistance have been essential for survival.
3. Inter-relationships: *Umunthu* provides a pragmatic framework for the relationship between the individual and the collective. Within this framework, there is no space for dissecting life into fragmented pieces but to realise life in inclusion. This realisation of inclusion does not facilitate an understanding of mental health as a separate concept, but as an integral part of wellbeing of the individual that exists within the collective.

Unsurprisingly with a philosophy so central to the culture of the world’s oldest human civilisation, it is impossible for any scholar to convey every characteristic and component of *Umunthu,* not least due to its essentially oral tradition and lack of written commentaries (Nussbaum 2003). However, this examination of *Umunthu* within interconnectedness, inclusion and inter-relationships, highlights the collective agency that supports the individual and where the operationalising of separate ‘mental health interventions’ represent unfamiliar terrain.

The facilitation of compassion, reciprocity, dignity, and mutuality is an internal process within the individual to realise that we share a common humanity. A person who possesses *Umunthu* attitude is capable of compassion, reciprocity, mutuality and caring for their fellow human beings without discrimination (Goduka 2000). In this understanding, the realised person of *Umunthu* is capable of dealing with external challenges of life including gender, class, social structures, disasters, conflicts or uneven development. This goes beyond the social, political, economic and cultural structures of the community.

Although an ancient philosophy, *Umunthu* also represents a contemporary approach to living (Bandawe 2010). And while, as in any culture, there are traditional lines of thinking and expression, these are continually influenced by wider social changes, such as from globalisation or moves to urbanisation, which demand a re-examination of people’s lives, of their relationships with others and their environment. Culture is “neither homogenous, nor determinative nor unchanging” (Kleinman 1995:58) and active ‘cultural engagement’ has been shown to shed light into challenges of contested issues like gender, age and illness. Scholars such as du Plessis (2019) for instance have demonstrated that *Umunthu* provides a community-centred and collective sense of care, which can be operationalised into dealing with gender-based challenges, with principles of *Umunthu* being incorporated into practical programming to prevent gender-based violence and improve human security (du Plessis 2019). Similarly, Benhabib (2003) and Masolo (2010) have shown the potential of *Umunthu* as a philosophy beyond mental health and wellbeing to assist with developmental challenges such as urbanisation and globalisation where the lives of communities can be positively transformed.

## *Umunthu*: Understandings of Mental Health and the HSAs’ Interventions

As a ‘life force’, it can be seen that every living person possesses *Umunthu,* and this is realised within their own internal capacities. While there are some differences of interpretation as to whether a person can be alive without it, one strand of traditional thought in Malawi suggests that mental illness temporarily affects *Umunthu* (Steinforth 2009). “*Mzimu wa umunthu*” literally means a de-socialisation where a person retains their vital life force but no *Umunthu*. However, a person’s *Umunthu* can be redeemed dependent on the cause of the current difficulty. Any mental disorder can be attributed to a number of different causes from social factors to internal functions. In traditional medicine in Malawi, a diagnosis is achieved once the traditional healer (a *sing’anga*) has identified the origin (or culprit, if bewitchment is suspected), the affected person takes prescribed potions, and the person or family have undertaken the required ritual. If the cause is diagnosed from moral transgression (going against traditional *miyambo*), then the traditional healer will advise on particular combinations of herbs, potions and rituals. If, however, the origin of the mental illness is considered God’s will, then this may be seen as more difficult to treat (and ‘palliative’, since no man can overturn God’s will) but nevertheless re-gaining a person’s *Umunthu* remains the goal. For example, in situations of epilepsy (*khunyu*), thirty different remedies are variously effective (Steinforth 2009).

Clearly, living communally and holding as vital particular ways that a person should behave in his or her personification of *Umunthu* is a significant factor that differentiates perceptions of effective treatment from much western and biomedical approaches where the individual is treated. Traditional healers treat the whole person/family/community in a collective healing process. Indeed since ‘formulations’ include understanding and identifying the source of the problem, a person is not considered treated until having undertaken traditional rituals that satisfy social requisites (Kirmayer 2004; Steinforth 2009).

In the same way, the practical interventions the HSAs employed to engage and mobilise support through the existing traditional and community structures appear to access the ‘moral economy’ (Scott 1979) of the community. The traditional systems of exchange, reciprocity and social insurance are accessed and underpinned by *miyambo* on an individual and collective basis. As a result, through village chiefs, clan-based distribution and village health committees, HSAs were able to respectably and successfully access patronage for their patients in the form of protection, food, shelter, money and the assistance of family or community elders, with the positive benefit this will have to their ‘mental health’.

Within such a setting, the HSAs’ intervention of counselling too becomes adapted to its cultural context. A person in distress is looking for a way to relieve their distress and—in the European and North American culture from which humanistic psychotherapy has derived—this is seen as most effectively achieved through a non-judgemental non-directive approach whereby a person generates their own solutions to their difficulties (Rogers 1961). However, in less individualistic cultures, the solution may be better informed through a reminder of his or her place within the community and reinforced by the importance of *miyambo* as a way of re-gaining *Umunthu*. The fact that U*munthu* fundamentally involves behaving in a way that promotes the harmony, and wellbeing of the group informs this more directive form of counselling that the HSAs employed. Hence, within the community, the HSAs hold and are perceived to hold a certain knowledge and expertise around ‘health’ means that they are obliged (through *Umunthu*) to share this wisdom and advice. For within the collective cultural setting, there is an individual responsibility and duty towards everyone within the community, which allow the HSAs to advice, guide and directly be involved in the lives of the people that are seeking help. Of course, this is a messier and more complicated affair than the individualistic counselling model proposed by Rogers (1961). Normal and shared practices of this type are so troubling to ‘outsiders’ precisely because what looks so wrong from the outside, does not look that way from ‘within’—from the perspectives of HSAs and their patients (Kleinman 2006).

Finally, a commonly mistaken stereotype in such cross-cultural comparisons is the notion that, given the communal inclination of a Malawian, their treatment for mental illness needs to involve collective or group approaches. While a surface understanding of *Umunthu* accurately points to the general command that it values communal notions of living in contrast to the alternative individual-centric western approach, and therefore obliges group and community support responses—‘convenient’ also where resources are limited! A closer study of *Umunthu,* however, reveals a far more sophisticated and multifaceted philosophy that never loses sight of the individual in their relationship with others. Wilson and Williams (2013) note that in Akan philosophy originating from West Africa, the ontological completeness of the individual is centred in the appreciation of his/her obligation to members of their group while his/her individuality is in no way displaced by the collectiveness of the group (Gyekye 1996). Thus, although HSAs established many group interventions—health promotion and support groups—the tight facilitation, didactic presentation and scrupulous respect for hierarchies of age, gender and community status made the events very different from the egalitarian/person-centred and demonstrably challenging support groups in western societies. Yet again, such styles of facilitation and group processes reflect the nature of *Umunthu* and what it is to be simultaneously offering and receiving support in a collectivist culture. Crucially, since it is through our own cultural lens that we see and interpret the world, gain our understandings of mental illness and how best to respond, any effective interventions must draw on these substantive and nuanced understandings.

## What Can We Learn from These Experiences in Malawi?

MGMH approaches to mental health problems can provide value, as evidenced in many parts of the world, but through closer examination of a specific task-shifting initiative, this paper has begun to expose the integral assumptions operating within the therapeutic interactions between patient and health worker. Adopting an exclusively biomedical framework around the causes, experience of and response to distress when people’s cultural lens differs risks alienating the ‘distressed’ from the helper. Future exploration with HSAs themselves would elaborate further the nature and properties of the interventions they are employing, through which it is envisaged adaptations, derivations and “often contradictory and inconsistent” experiences will expose new ways to understand and provide support for people in distress (Siddiqui, LaCroix and Dhar 2014:294).

For while what drives the universalistic assumptions about effective responses to distress has been examined and critiqued for two decades, the explanation for why local health workers such as HSAs act in this way may be equally revealing. Mills (2014) speaks of the ‘pretending’ of patients confronted with the power of the psychiatric gaze—and links this to the mimicry and subversion of the colonised. Perhaps the same is happening here for HSAs: they are operating a ‘sly normalisation’ and ‘invisibility’, whereby what may be heralded as biomedical interventions are in fact the delivery of culturally embedded therapeutic approaches. For the HSAs, practical help is marshalled through traditional structures, counselling becomes advice to provide the person hope and a steer, and support groups become more than providing a safe place for emotional exchange, to a forum for the enactment of values, reflection and reinforcement of *Umunthu*; of positive social capital and obligation.

These responses are entirely functional and embedded in the language and culture in which Malawians operate, and, just as in other societies and cultures, they derive from a rich seam of indigenous philosophy and knowledge that represent ways to promote wellbeing and personhood so that collective norms and values can survive in a globalised world. Interestingly, Mills has recently noted a similar departure from theory to enactment in mhGAP interventions in India (Mills and Lacroix 2019).

Indeed, this recognition of difference is vital for any ‘movement’ seeking to extend a reach across the globe. As this paper reminds us, communities around the world provide a large treasury of knowledge and understandings that need to be not only acknowledged but also engaged with. The alternative risks a repeat of the colonial endeavour. For while from a political and ideological perspective, the membership and structure are somewhat different, there are parallels between the MGMH and the colonial project where, originating from a European context, local societies, cultures and most importantly knowledge systems are looted and destroyed. The discrediting of the ‘local’ and replacing with the ‘scientific’ mimic colonialism (Fernando 2014), as people are cutoff from indigenous knowledge and agency.

The statement by Macaulay—the British politician and historian, dated the 2nd February 1835—sums-up parallels between the MGMH and the colonial project. His words also unwittingly alert us to the chilling danger of producing certain types of ‘interpreters’:

“I have never found one among them who could deny that a single shelf of a good European library was worth the whole native literature of India and Arabia. … neither as the languages of law nor as the languages of religion have the Sanscrit and Arabic any peculiar claim to our encouragement. We must at present do our best to form a class who may be interpreters between us and the millions whom we govern,—a class of persons Indian in blood and colour, but English in tastes, in opinions, in morals and in intellect.”

(Macaulay 1835).

From: Bureau of Education. Selections from Educational Records, Part I (1781–1839).

From a political ecology perspective, Macaulay’s statement explains the way that the colonial project shatters the relationships between social, political, cultural and economic structures and the environment of the colonies (Castro-Gomez 2003; Said 1994; Mudimbe 1988). Similarly, the biomedical approach to health facilitates a strong disconnection between the human systems and the environment (McLeod 2000; Brown 1985; Illich 1976), instead installing a particular epistemology and frame.

An incident during a workshop with different stakeholders in Malawi facilitated by the first author (JW) to design the bespoke HSA mental health curriculum illustrates the same assumed status of different epistemologies and the embodiment of a new ‘interpreter’ role by local mental health professionals. Traditional Chewa culture includes a number of *miyambo* that reflect the *Umunthu* philosophy of respect for personhood instructing people not to ridicule or denigrate people with disabilities. Traditional proverbs encourage the social integration of people with mental health problems and even ascribe particular value and competence (Steinforth 2009). However, a suggestion to refer to such instruction as a part of anti-stigma messages within the HSA curriculum was immediately dismissed by all mental health professionals present as not suited to an evidenced biomedical position. It was clear that a local protective measure for the disabled was being disparaged and dismissed, with the loss of a potential bridge between epistemologies. At the same time, the mental health professionals openly expressed concern that their own status, and that of the HSA mental health curriculum, would be negatively perceived if it was in any way associated with traditional ways of thinking. So, whilst genuine and heartfelt, the health professionals’ concern for patients was being expressed through the modernising mission or colonising of biomedicine and silencing of traditional knowledge (Ibrahim 2014).

For what is important here was the lack of room for discussion and debate. Biomedicine is underwriting a process of cultural change and devaluation of traditional instructions without consideration that “services (should) reflect the beliefs and practices of local people” (White and Sishidharan 2014:416). Instead MGMH terminology and classifications are obscuring indigenous philosophies, ways of understanding distress and helping approaches, of which *Umunthu* is just one example. By contrast in the real world, in communities, HSAs do not have the luxury of adopting learned or elevated positions, and instead use their versions of psychosocial interventions to engage with problems as their community sees them and drawing on culturally embedded knowledge and approaches to inform their support for distressed people. Here, *Umunthu* is presented, not as an unqualified elucidation of a philosophy for positive mental health, but simply as the cultural framework underpinning the lives of local people. If MGMH approaches are to live up to their promise of promoting mental health and reducing suffering across the world, then a dialogue needs to happen in a manner that shows a willingness to collaborate as equal partners (Kirmayer and Pederson 2014; Campbell and Burgess 2012) where questions are asked rather than always providing answers.

While attempts to widen the range of scientific disciplines within the MGMH evolution have been evident (Bemme and Kirmayer 2020) and are reflected in part by the emergence of particular psychosocial interventions in publications like the *mh*GAP intervention guide (WHO 2016), it nevertheless retains a disease-orientated model with psychocentric and related therapeutic assumptions. Instead a meaningful and concerted approach to understand the fundamental philosophical basis and cultural norms of a community, based on humility, openness and respect, would provide the platform for a different kind of ‘movement’ that would demonstrate a more democratic and inclusive dialogue from which to develop responses that contribute rather than depreciate or eclipse local indigenous healing.

## Conclusion

This article has demonstrated how HSAs have responded to people’s distress with psychosocial interventions that are attuned to their patients’ cultural framework of *Umunthu* and have done so under the gaze of a biomedically informed mental health initiative. For the person in distress and the HSA, their sense of being cannot be separated from their own sense of personhood within the family, community and social context. The authors acknowledge the contribution of the biomedical model and appreciate that the individualistic clinical model of psychological care provides helpful insights and directions. However, the argument here is that the biomedical model is demonstrably not the only model of care informing mental health interventions yet it has become the authoritative understanding of distress deriving from all uncertainty and dangers in life. Like the colonial project, it assumes superiority to all the other models of care and its domination functions on different levels and through various methods that focus on the individual without understanding the social, political, cultural, economic and environmental contexts. As a frontline organisation spreading this ‘gospel’, the MGMH has the capacity not only to form a class of “interpreters” but also to destroy the existing philosophies and knowledge systems that remain rooted and relevant to local populations. Resisting such destruction and preserving and mobilising these indigenous understandings become a question of social justice (Croft et al. 2016). For, as Bertolote (2008) argues, mental health is more than a scientific discipline and instead has strong political and ideological origins that seek to promote human rights and quality care for all. In this, a truly respectful collaboration as equal partners with village health workers, traditional healers, faith practitioners and witch doctors is possible. Such collaboration will allow the ‘external’ biomedical practitioners and ‘internal’ healing practitioners to learn from each other and together develop responses to distress that are most effective and relevant to the populations they serve.
